# Super delayed phase imaging in gadoxetic acid-enhanced MRI: investigating factors contributing to improved liver contrast

**DOI:** 10.1007/s00330-024-11227-z

**Published:** 2024-11-29

**Authors:** Tomohiro Kobayashi, Kazuto Kozaka, Takashi Matsubara, Akira Yokka, Saya Igarashi, Azusa Kitao, Norihide Yoneda, Miho Okuda, Toshifumi Gabata, Osamu Matsui, Satoshi Kobayashi

**Affiliations:** 1 Department of Radiology, Kanazawa University Graduate School of Medical Sciences, Kanazawa, Japan; 2 Faculty of Health Sciences, Institute of Medical, Pharmaceutical and Health Sciences, Kanazawa University, Kanazawa, Japan

**Keywords:** Hepatocellular carcinoma, Liver function, Super delayed phase imaging, Liver-to-spleen contrast

## Abstract

**Objectives:**

To assess whether extended delayed phase imaging, performed after gadoxetic acid administration for 60–120 min (termed as super delayed phase [SDP]), improves liver contrast and nodule visibility in patients with chronic liver disease and to identify predictors for contrast enhancement.

**Methods:**

In this retrospective study, 116 patients with chronic liver disease were selected from 6933 gadoxetic acid-enhanced MRI examinations, which included SDP images. The liver-to-spleen contrast (LSC) was quantitatively evaluated, and factors influencing the improvement of LSC were analyzed. By comparing the standard hepatobiliary phase images at 20 min post-contrast (HBP20) with SDP images, nodule visibility was evaluated by two readers who were blinded to the study.

**Results:**

SDP significantly enhanced LSC (SDP: 1.81 ± 0.48 vs HBP20: 1.50 ± 0.34, *p* < 0.001) and improved nodule visibility in patients with initially poor LSC. Total bilirubin levels and visible biliary excretion during HBP20 are predictors of LSC enhancement. Furthermore, nodule visibility scores significantly increased in the group with poor initial contrast (Reader 1: from 2.92 ± 1.57 to 3.79 ± 1.44; Reader 2: from 2.34 ± 1.42 to 3.36 ± 1.57, *p* < 0.001).

**Conclusion:**

SDP enhanced liver contrast and nodule detection in patients with chronic liver disease, particularly in those with impaired liver function. Total bilirubin levels and visible biliary excretion during HBP20 are useful predictors of improvement. This technique may improve the diagnostic utility of MRI for hepatocarcinogenesis in cirrhotic nodules, specifically for detecting precursors of hepatocellular carcinoma, in cirrhotic patients with compromised liver function.

**Key Points:**

***Question*** *In gadoxetic acid-enhanced MRI, inadequate liver contrast can occur in patients with impaired liver function, potentially limiting the diagnostic value of the examination*.

***Findings*** *SDP images improved liver parenchymal signal intensity and visibility of hepatocellular carcinoma, even in cases with impaired liver function*.

***Clinical relevance*** *The addition of SDP imaging in gadoxetic acid-enhanced MRI improves liver contrast and early detection of hepatocellular carcinoma, especially in patients with impaired liver function, such as Child–Pugh B or C, aiding in making appropriate treatment decisions*.

**Graphical Abstract:**

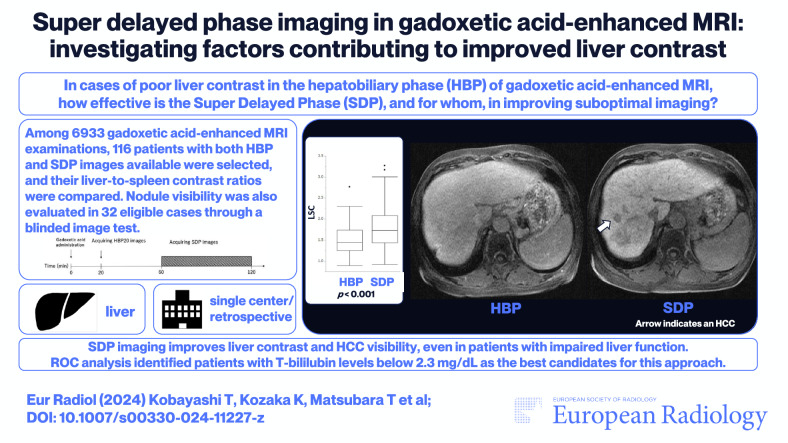

## Introduction

Hepatocellular carcinoma (HCC) is the most common primary malignancy of the liver and ranks third among cancer-related deaths worldwide [1]. Because HCC diagnosis in at-risk patients can be achieved noninvasively through imaging analysis, precise imaging diagnosis remains a critical clinical challenge. Gadoxetate disodium (Primovist or Eovist; Bayer Healthcare) contrast agents have been instrumental in hepatobiliary MRI [2–4]. Owing to its ability to highlight lesion-to-liver conspicuity in hepatobiliary-phase images (HBP), gadoxetate disodium is effective in detecting both advanced HCCs and borderline lesions such as high-grade dysplastic nodules and early HCC [2–10]. However, in some instances inadequate image contrast results from poor gadoxetic acid uptake by the liver parenchyma. Notably, patients with cirrhotic livers may exhibit diminished signal intensity in the HBP, assessed using the liver-to-spleen contrast (LSC), where a value below 1.5 is generally considered inadequate [11–13]. Because patients classified under Child–Pugh class B or C exhibit poor LSC [14], indications for gadoxetic acid-enhanced MRI should be carefully considered in clinical practice.

To extend the benefits of the excellent nodule detection capabilities of gadoxetic acid-enhanced MRI (not only for small lesions but also for borderline lesions) to more patients, methods are being explored to increase the liver contrast, even in cases with impaired liver function [15–19]. Among these, delaying the timing beyond 20 min for additional HBP imaging is one approach [18, 19]. This intuitive strategy extends the examination time to achieve sufficient hepatocellular uptake of the contrast agent; however, the burden of additional imaging remains a challenge. Consequently, the delayed timing was usually set at 30–60 min post-injection in previous studies [17, 18], and the efficacy of further delayed imaging has not yet been assessed. Although the 2018 version of the Liver Imaging Reporting and Data System Frequently Asked Questions (LI-RADS v2018 FAQ) reports that this approach may improve image quality in cirrhotic livers with diminished function, its impact on diagnostic accuracy remains unclear [20].

Therefore, this study aimed to (1) assess the effectiveness of acquiring additional images at extended intervals—specifically, from 60 min to 120 min after administering gadoxetic acid (referred to as the “super delayed phase” [SDP]) in patients with chronic liver disease and varying degrees of hepatic dysfunction and to examine the factors contributing to improved liver contrast; and (2) investigate whether the inclusion of SDP images enhances nodule visibility in cases with suboptimal liver contrast.

## Materials and methods

### Patients

**Fig. 1:**
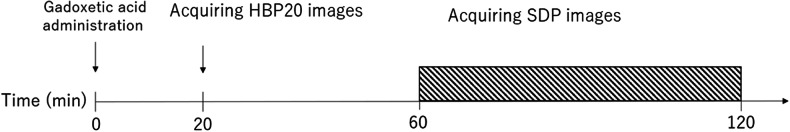
Imaging protocol with gadoxetic acid-enhanced hepatobiliary phase (HBP20) and SDP acquisition. The HBP20 image is acquired 20 min after the administration of gadoxetic acid. The decision to proceed with SDP imaging was determined by the radiologic technologist and radiologist. In the interim, one or two additional examinations may be performed before acquiring the SDP image. The timing of the SDP was defined as either less than 60 min or more than 120 min after gadoxetic acid administration in this study

**Fig. 2:**
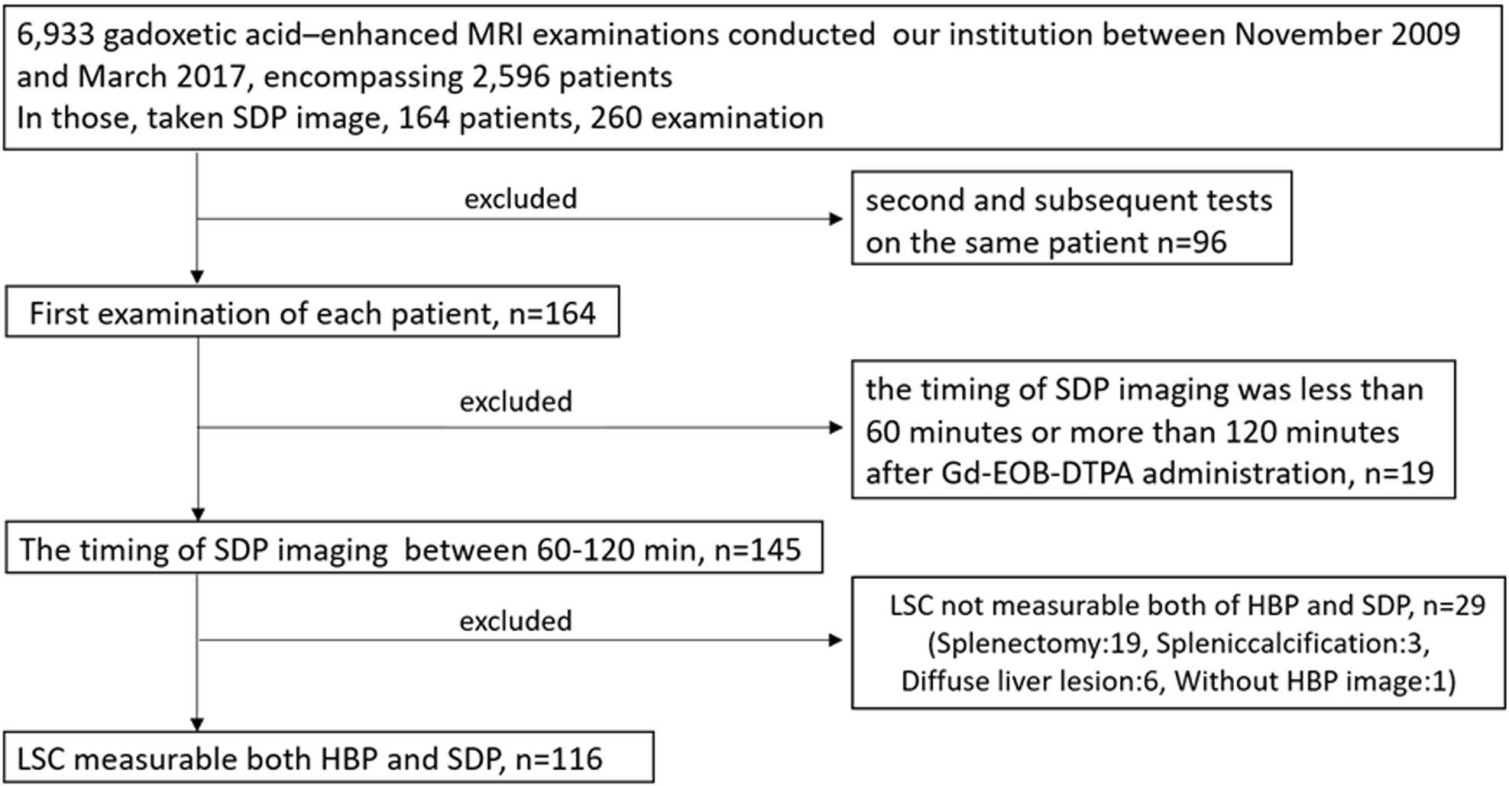
Study patient's flow chart

This retrospective single-center study was approved by the ethics committee of Kanazawa University Hospital. The requirement for written informed consent was waived because of the retrospective study design. Among the 6933 gadoxetic acid-enhanced MRI examinations performed at our institution between November 2009 and March 2017, which included 2596 patients, those that underwent additional delayed phases beyond our standard protocol were extracted for further analysis. Additional delayed imaging is performed in our facility only if the liver contrast is insufficient compared to that of the spleen or portal vein at the 20-min hepatobiliary phase (HBP20). The decision for extended imaging was made by the attending radiologist and technician, and SDP images were obtained only with patient consent. Typically, extended imaging is performed after a gap, usually involving one or two other examinations (Fig. 1). This practice is uncommon in other facilities and is crucial for understanding the “additional delayed phases” referred to in this study. The exclusion criteria were as follows: second and subsequent MRI examinations in the same patient; instances where the timing of the additional delayed imaging was < 60 min or > 120 min after gadoxetic acid administration; and patients in whom LSC could not be accurately measured in both HBP20 and SDP for reasons such as post-splenectomy state, splenic calcification, diffuse liver mass lesions, or HBP20 was not available (Fig. 2).

### Imaging technique

All individuals underwent a dedicated gadoxetic acid-enhanced MR imaging protocol tailored specifically for detecting and diagnosing HCC, using either a 1.5- or 3-T MR system with an eight-channel anteroposterior phased-array surface coil (Signa HDxt; GE Healthcare). The protocol included chemical-shift T1-weighted imaging, gadoxetic acid-enhanced dynamic study, fat-suppressed T2-weighted imaging, diffusion-weighted imaging (with *b*-values of 0 and 800), and HBP20. For the dynamic study, each patient received an intravenous bolus injection of gadoxetic acid (Primovist; Bayer Schering Pharma) at a dose of 0.025 mmol/kg body weight and a flow rate of 1 mL/s, followed by a 20-mL saline flush. The arterial, portal, and transitional phase imaging were performed using a fat-suppressed 3D SPGR T1-weighted sequence with the following parameters: LAVA-XV, TR/TE = 3.2–4.0/1.6 ms, flip angle 6–15°, field of view 42 × 42 cm, matrix 192 × 320, interpolated to 512 × 512, slice thickness 4.2 mm, and overlap 2.1 mm. HBP20 was obtained 20 min after the injection of the contrast material. If liver contrast was determined to be inadequate by the concerned specialist, additional SDP imaging was performed after the completion of another examination.

### Quantitative assessment of LSC

Oval-shaped regions of interest (ROIs) were placed in the right lobe of the liver and the spleen at the level of the porta hepatis during both the HBP20 and SDP intervals by a board-certified radiologist with an experience of 7 years (T.K., blinded to the study). The liver ROI was situated approximately 1 cm from the liver surface, carefully avoiding focal lesions and major vascular branches. The spleen ROI was set approximately 1 cm from the splenic surface. The LSC was calculated for every case of both HBP20 and SDP. Based on a previous study [11–13], an LSC index of > 1.5 and ≤ 1.5 was defined as good and poor LSCs, respectively. Among the patients with poor LSC in HBP20, those that turned to good LSC in SDP were categorized as “improved,” whereas those LSCs that remained poor were categorized as “non-improved.”

The ΔLSC was defined using the following equation:

$$\Delta {{\rm{LSC}}} = {{\rm{LSC}}}\; {{\rm{in}}}\; {{\rm{SDP}}}-{{\rm{LSC}}}\; {{\rm{in}}}\; {{\rm{HBP}}}20$$

### Visual contrast excretion into the bile duct

The presence or absence of contrast agent excretion into the bile duct during HBP20 was independently examined by two radiologists who were blinded to the study (A.K., with 21 years and T.K., with 7 years of experience). In cases of disagreement, consensus was reached through discussion.

### Evaluation 1: analysis of clinical and radiological markers for predicting changes in LSC (ΔLSC) and improvement in SDP

To investigate clinical and radiological markers correlating with changes in LSC (ΔLSC), we studied the relationships between ΔLSC and various factors, including the Child–Pugh classification class, prothrombin time (%), albumin level (in g/dL), total bilirubin level (in mg/dL), presence of ascites, encephalopathy, and the correlation of ΔLSC with visual contrast excretion in the bile duct. Subsequently, we focused on patients exhibiting poor LSC in HBP20 and analyzed these markers to determine predictors for classification into the improved group.

### Evaluation 2: blinded image interpretation test on nodule visibility

A reading test focusing on nodule visibility was conducted in a cohort of patients with poor image quality. Patients with a maximum of five nodules with diameters ranging between 5 mm and 30 mm and those patients with nodules categorized as LI-RADS 4–5 were enrolled. Two independent readers (A.K., 21 years, Y.A., 9 years, both blinded) reviewed both the SDP and HBP20 images, presented as single cross-sectional slices containing nodules, in random order, and rated the visibility of each nodule on a 5-point scale: 1 (not visible), 2 (barely visible), 3 (intermediate visibility), 4 (visible), and 5 (clearly visible). The visibility scores of HBP20 were compared with those of SDP.

### Statistical analysis

Patient characteristics data are presented as median with interquartile ranges or means ± standard deviations. Data normality was checked using the Shapiro–Wilk test. The LSC values of HBP20 and SDP cells were compared using a paired *t*-test. ΔLSC between Child–Pugh groups were analyzed using the ANOVA followed by Steel–Dwass post hoc. The relationship between ΔLSC and Child–Pugh criteria was examined using logistic regression and Pearson’s correlation analyses. The association between bile duct contrast agent presence and ΔLSC was analyzed with the student’s *t*-test, whereas its relation to SDP timing was analyzed using the Mann–Whitney *U*-test. The inter-rater agreement in image interpretation was determined using the weighted kappa coefficient and visibility using the Mann–Whitney *U*-test. Differences between the improved and non-improved groups were assessed using the chi-square and *t*-tests. Multiple logistic regression analyses were performed on factors for which significant differences were observed between the two groups. All statistical analyses were performed using Python 3.8. Statistical significance was set at *p* < 0.05.

## Results

We enrolled 116 patients who had undergone MRI examinations. The median time from the injection of the contrast agent to the acquisition of the SDP images was 80.0 min [range, 60–120 min]. Table 1 summarizes the patients’ characteristics, Child–Pugh classification, each factor, and MRI settings. Most patients (99/116, 85.3%) had viral hepatitis, alcoholic hepatitis, nonalcoholic hepatitis, or a combination of these. Additionally, 98 of the 116 patients (84.5%) were diagnosed with cirrhosis.

**Table 1:** Patient characteristics, laboratory data, and MRI settings

Patient characteristics	value
Sex, male/female	67/49
Patient age [median years (IQR)]	68 (62–75)
Background liver disease	
HCV-Ab positive^a^	57
HBs-Ag positive^a^	10
Alcohol^a^	12
NASH	7
Others	17
Cirrhosis (yes/no)	98/18
Laboratory data	
Child–Pugh classification (A/B/C), (*n* = 104)	41/47/16
PT [%, median (IQR)], (*n* = 104)	68 (58–78)
Total-bilirubin [(mg/dL, median (IQR)], (*n* = 108)	1.5 (1.0–2.2)
Albumin [g/dL, median (IQR)], (*n* = 108)	3.2 (2.8–3.7)
Ascites (absent/mild/moderate to severe)	84/25/7
Hepatic encephalopathy (none/grades 1 to 2/grades 3 to 4)	110/6/0
MRI setting^b^	
Magnetic field strength, 1.5 T/3.0 T	48/68
Slice thickness (gap)^b^, mm	4.2 (2.1)
TR (ms)/TE (ms)/flip angle, (degree)^b^	3.2–4.0/1.6–2.3/12–15
Delayed time of SDP [(min, median (IQR)]	80 (70–91)

*IQR* interquartile range, *TR* repetition time, *TE* echo time, *SDP* super delayed phase

^a^ Four patients were double positive for both HCV-Ab and HBs-Ag, seven for HCV-Ab with a history of heavy alcohol intake, and two for HBs-Ag with a history of heavy alcohol intake

^b^ All hepatobiliary phase and SDP images were captured by 3D gradient echo sequence (LAVA-XV)

**Fig. 3:**
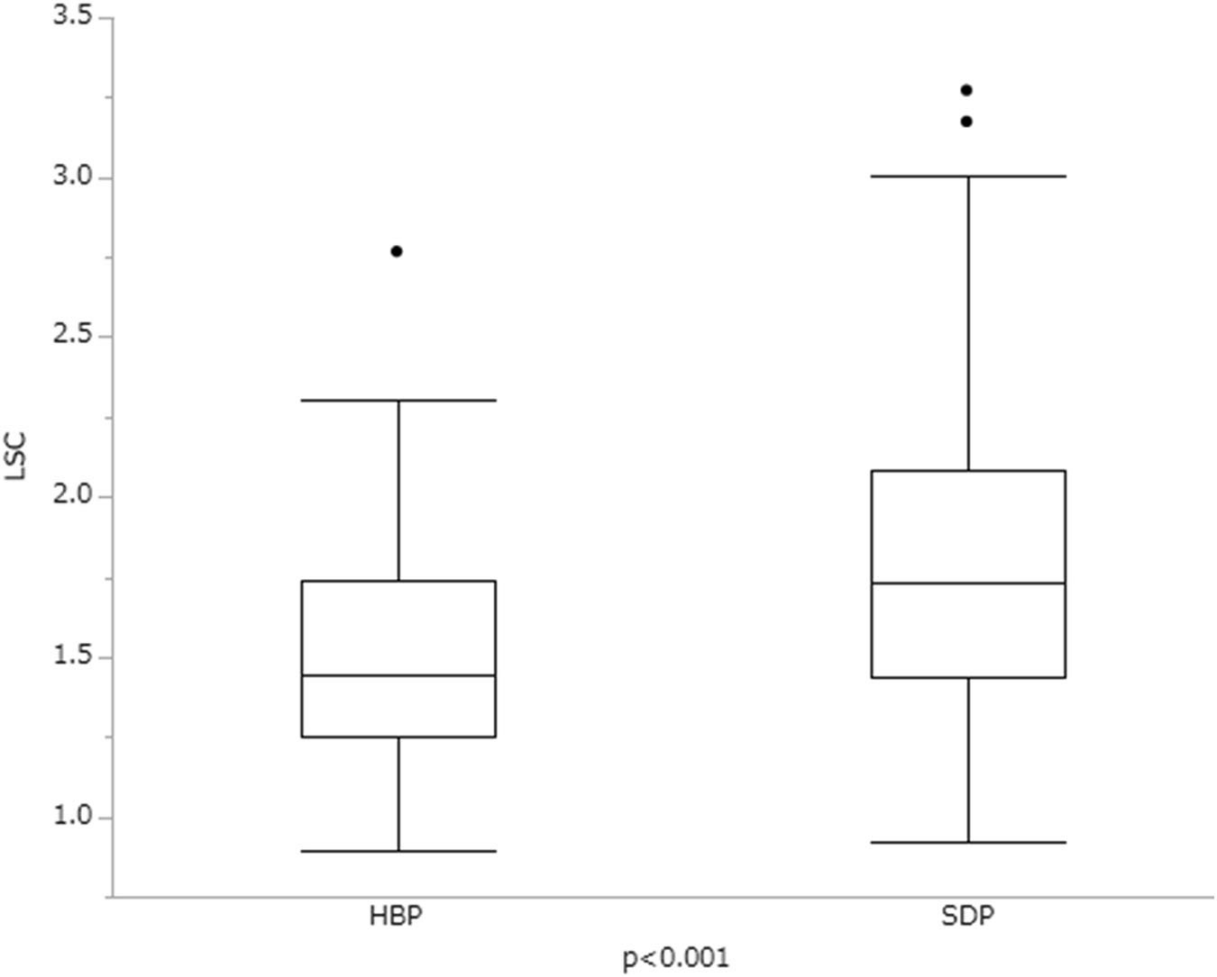
Comparison of liver spleen contrast (LSC) at hepatobiliary phase (HBP20) and SDP. The graph demonstrates statistically significant differences in LSC between the HBP20 and the SDP, with LSC at HBP being 1.50 ± 0.34 compared to 1.81 ± 0.48 at SDP (*p* < 0.001)

The average liver and spleen SI for both HBP20 and SDP was as follows: for HBP20, liver SI was 561.9 ± 232.7, spleen SI was 379.1 ± 146.8, and for SDP, liver SI was 587.5 ± 235.5, with spleen SI at 335.6 ± 135.8. The LSC in SDP was significantly higher compared to that in HBP20 (1.81 ± 0.48 vs 1.50 ± 0.34, *p* < 0.001) (Fig. 3), and the ΔLSC showed a weak positive correlation with SDP acquisition time (a correlation coefficient of *r* = 0.288 [*p* < 0.01]). Positive ΔLSC values were recorded in 98 of the 116 patients (84%).

Of the 116 analyzed patients, 68 (58.6%) had poor LSC in HBP20. Among the 68 patients with poor LSC, 37 (54.4%) improved to good LSC in SDP, and 31 (45.6%) remained poor LSC.

### Evaluation 1: analysis of clinical and radiological markers for predicting ΔLSC and improvement in SDP

**Fig. 4:**
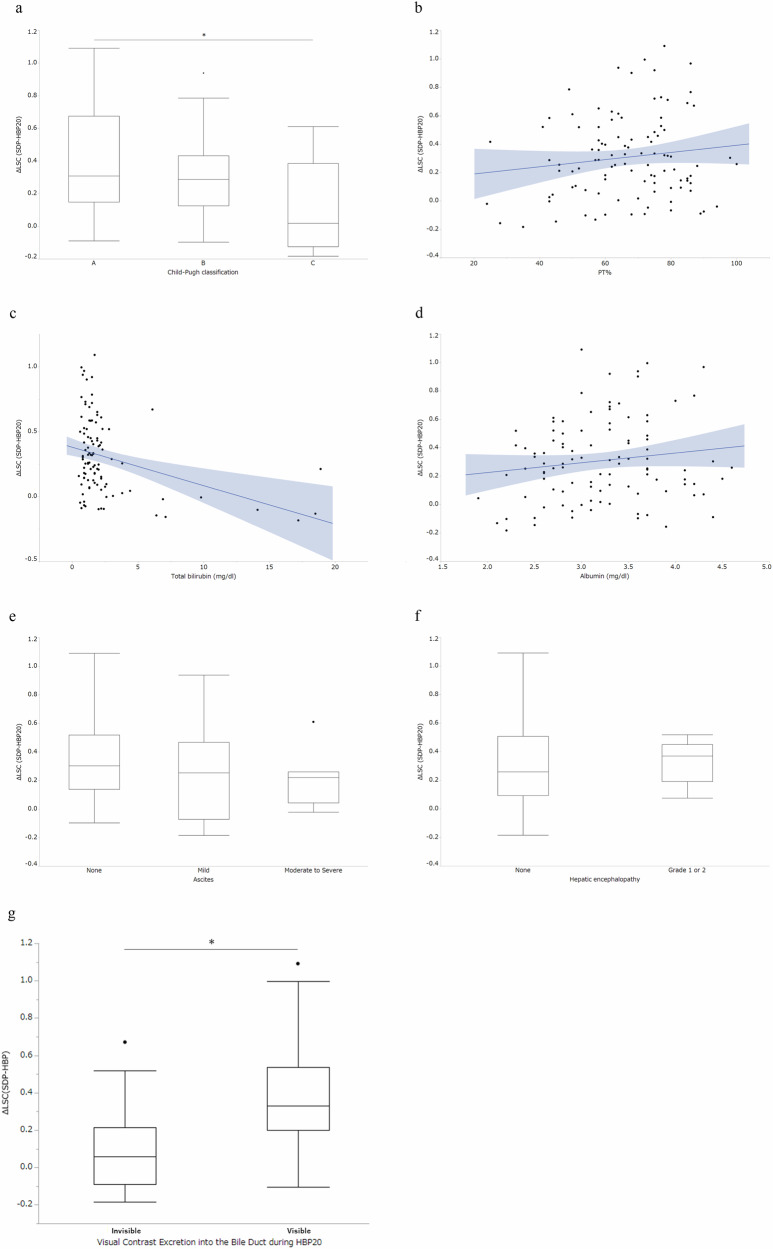
Relationship between Δliver spleen contrast (LSC) and Child–Pugh classification, its component factors, and visual biliary excretion. **a** ΔLSC vs Child–Pugh classification, (**b**) ΔLSC vs prothrombin time (PT), (**c**) ΔLSC vs total bilirubin, (**d**) ΔLSC vs albumin, (**e**) ΔLSC vs ascites, (**f**) ΔLSC vs hepatic encephalopathy, (**g**) ΔLSC vs visual biliary excretion. Figures **a**, **e**, **f**, and **g** are represented as box plots, which display the median, interquartile range, and outliers. The remaining figures are scatter plots, each featuring a trend line with a shaded area representing the 95% confidence interval. An analysis of variance revealed a significant difference in ΔLSC among the Child–Pugh classifications, Child–Pugh A: 0.39 ± 0.32, Child–Pugh B: 0.30 ± 0.23, and Child–Pugh C: 0.13 ± 0.27 (*p* = 0.009), and a statistically significant difference was discerned in ΔLSC values between the Child–Pugh A and Child–Pugh C groups (*p* = 0.006, ANOVA post-hoc Tukey-Kamar test) (**a**). ΔLSC demonstrates a weak negative correlation with total bilirubin (**c**), with no significant correlations noted with prothrombin time (PT) (**b**), albumin (**d**), ascites (none: 0.33 ± 0.28, mild: 0.25 ± 0.31, and moderate to severe: 0.21 ± 0.19) (**e**), or hepatic encephalopathy (none: 0.30 ± 0.29, grades 1 to 2: 0.33 ± 0.14) (**f**). A statistically significant difference was observed between patients with visible visual contrast excretion into the bile duct during HBP20 and those without (visible: 0.38 ± 0.27, invisible: 0.09 ± 0.20) (**g**)

An analysis of variance (ANOVA) revealed a significant difference in ΔLSC among the Child–Pugh classifications (*p* = 0.009) and a statistically significant difference was discerned in ΔLSC values between the Child–Pugh A and Child–Pugh C groups (*p* = 0.02). A weak negative correlation was observed between T-bilirubin and ΔLSC with respect to the Child–Pugh indices (*r* = −0.34, *p* < 0.001), whereas no significant correlations with ΔLSC were observed in other indices. Biliary excretion was visually observed in 86 patients (74.1%) and was not observed in 30 patients (25.9%) and ΔLSC values were significantly higher in the group with visible biliary excretion of the contrast agent at HBP20 (0.38 ± 0.27 vs 0.09 ± 0.20, *p* < 0.01) (Fig. 4). Table 2 summarizes the patient characteristics, laboratory data, and MRI settings of the improved and non-improved groups. Univariate analysis showed a statistically significant difference in three parameters, including T-bilirubin levels (*p* = 0.01), visual contrast excretion in the bile duct during HBP20 (*p* = 0.01), and delayed SDP time (*p* = 0.03) between the improved and non-improved groups. See the subanalysis regarding the effect of SDP timing on liver contrast improvement (electronic supplemental material). A multivariate analysis of these three parameters revealed that T-bilirubin levels and delayed time of SDP were independent factors (*p* = 0.03, *p* = 0.02, respectively), indicating that it is an independent parameter for improving LSC. Receiver operating characteristics (ROC) analysis identified 2.3 as the optimal T-bilirubin cutoff (AUC, 0.696; sensitivity, 0.969; and specificity, 0.467).

**Table 2:** Patient characteristics, laboratory data, and MRI settings of improved group and non-improved group

	Improve group, (*n* = 37)	Non-improved group, (*n* = 31)	Univariate analysis	Multivariate analysis
Patient characteristics				
Sex, male/female	15/22	20/11	*p* = 0.08	
Patient age, (years, mean ± SD)	69.1 ± 9.0	64.5 ± 11.4	*p* = 0.07	
Cirrhosis, (yes/no)	33/4	26/5	*p* = 0.08	
Laboratory data^a^				
PT (%, mean ± SD)	68.0 ± 14.0	61.3 ± 17.7	*p* = 0.11	
Total-bilirubin, (mg/dL, mean ± SD)	1.6 ± 0.9	4.2 ± 5.1	*p* = 0.01	*p* = 0.03
Albumin, (g/dL, mean ± SD)	3.1 ± 0.6	3.1 ± 0.5	*p* = 0.96	
Ascites, (absent/mild/moderate to severe)	28/8/1	17/10/4	*p* = 0.12	
Hepatic encephalopathy, (none/grades 1 to 2/grades 3 to 4)	33/4/0	30/1/0	*p* = 0.46	
MRI setting^b^				
Magnetic field strength, 1.5 T/3.0 T	13/24	11/20	*p* = 1.0	
Flip angle, 12°/15°	10/27	8/23	*p* = 1.0	
Delayed time of SDP, (min, mean ± SD)	88.9 ± 14.3	80.1 ± 14.3	*p* = 0.03	*p* = 0.02
Visual contrast excretion into the bile duct during HBP20, (positive/negative)	6/31	15/16	*p* = 0.01	*p* = 0.26

*IQR* interquartile range, *TR* repetition time, *TE* echo time, *SDP* super delayed phase, *HBP* hepatobiliary phase

^a^ For these cases, 60 had verifiable PT, 62 for total bilirubin and albumin. Ascites and hepatic encephalopathy status were confirmed for all

^b^ All hepatobiliary phases and SDPs were captured by 3D gradient echo sequence (LAVA-XV)

### Evaluation 2: blinded image interpretation test on nodule visibility

**Fig. 5:**
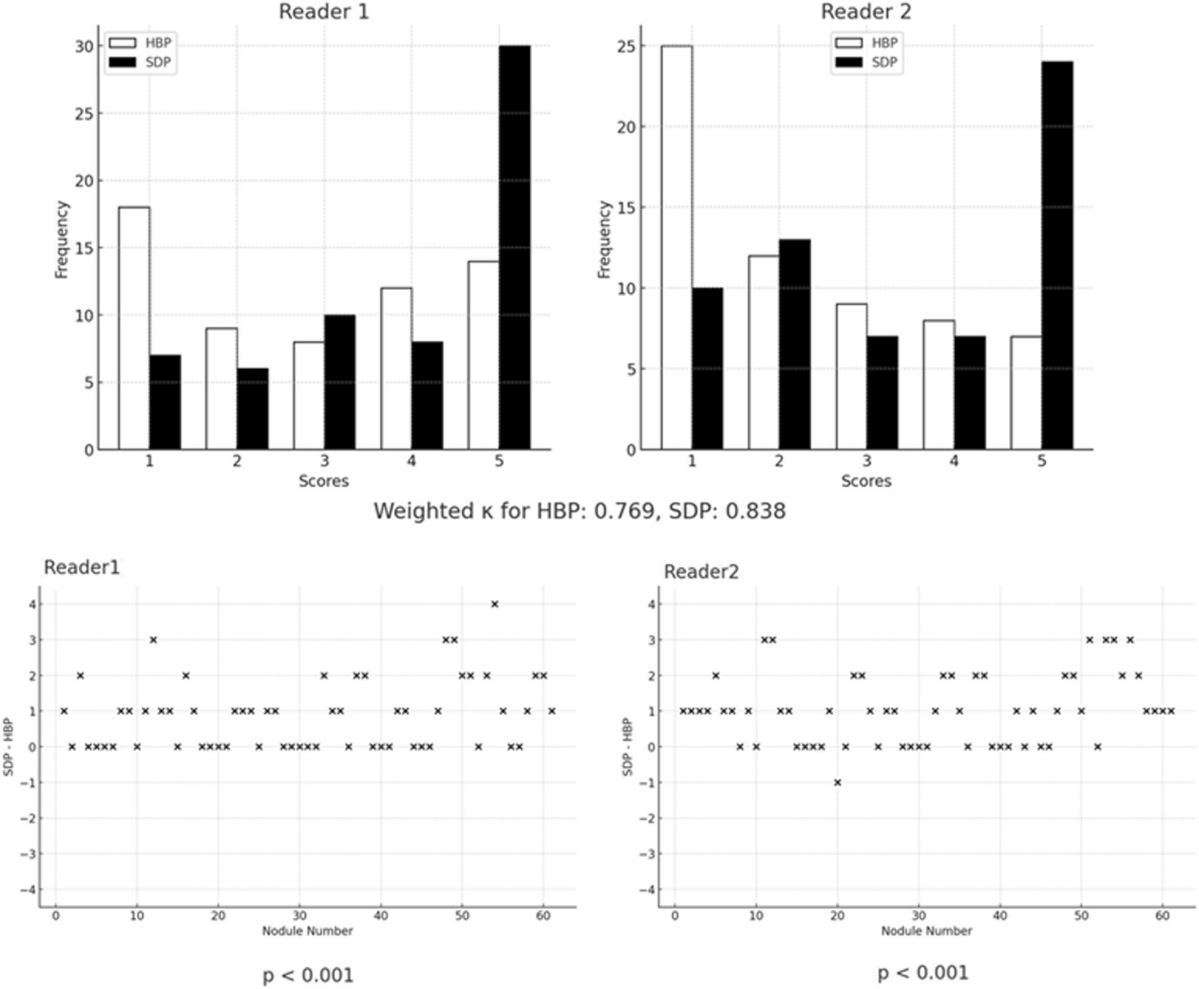
Results of the blinded reading assessment. Above column: quantification of each score (1–5) as allocated by individual readers. Below column: the difference between the SDP score and the hepatobiliary phase (HBP20) score corresponding to each nodule by each reader. Only one nodule showed a decreased SDP score (−1 decline) according to the evaluations by Reader 2. Reader 1 evaluated all nodules as unchanged or improved at SDP

**Fig. 6:**
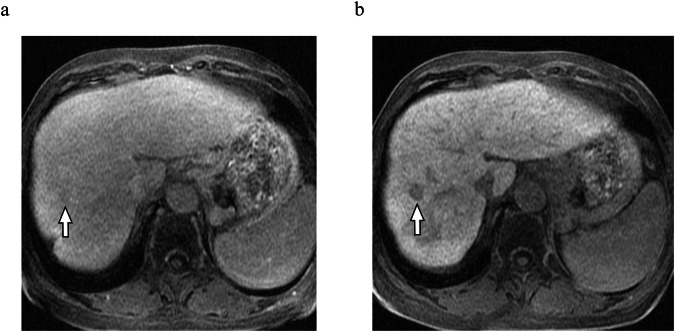
Representative case illustrated with a 71-year-old woman with hepatitis C virus-associated liver cirrhosis, Child–Pugh classification B (score 9). **a** hepatobiliary phase (HBP)20. **b** SDP images. SDP images taken 80 min after contrast administration show a remarkable improvement in nodule visibility in liver segment 8 (arrow). For this HCC, Reader 1 scored it as 2 during HBP20 and 5 during SDP, indicating a +3 improvement. Similarly, Reader 2 scored it as 2 during HBP20 and 4 during SDP, marking a +2 improvement

Of the 32 patients with poor LSC in HBP20, 61 nodules with an average size of 10.4 ± 4.7 mm were detected and assessed. Figure 5 shows the distribution of the readers’ scores for each nodule and a scatter plot of the score changes. The nodule visibility score was significantly higher in SDP than in HBP20 for both Reader 1 (2.92 ± 1.57 vs 3.79 ± 1.44) and Reader 2 (2.34 ± 1.42 vs 3.36 ± 1.57), with *p* < 0.001. The inter-reader agreement was quantified as weighted κ values of 0.769 (95% confidence interval [CI]: 0.637–0.866) and 0.838 (95% CI: 0.742–0.904) for HBP20 and SDP, respectively. A representative example is shown in Fig. 6. The scores for all nodules either remained stable or increased from HBP20 to SDP, according to Reader 1’s assessments. Similarly, Reader 2’s assessments revealed that the scores for most nodules either remained stable or increased, except for a single nodule that showed a score decrease of 1.

## Discussion

In this study, SDP imaging significantly improved liver parenchyma contrast in patients with chronic liver disease, as indicated by LSC metrics (SDP: 1.81 ± 0.48 vs HBP20: 1.50 ± 0.34, *p* < 0.001). Moreover, total bilirubin levels were negatively correlated. An optimal T-bilirubin cutoff of 2.3 mg/dL and visual contrast excretion during HBP20 proved to be simple and useful indicators for predicting improvement in patients with poor LSC in HBP20. A Blind Image Interpretation test revealed better visibility of hepatic nodules in the ‘poor’ LSC group using SDP (Reader 1: 2.92 ± 1.57 to 3.79 ± 1.44; Reader 2: 2.34 ± 1.42 to 3.36 ± 1.57, *p* < 0.001).

This study used two methods to evaluate the improvement in liver contrast provided by the addition of SDP images. The first method used a quantitative index, ΔLSC, which is the difference in LSC values between HBP20 and SDP. The second method assessed whether the LSC value exceeded 1.5, which is directly related to the visibility of liver nodules and is deemed clinically significant [11, 12].

Positive ΔLSC values were recorded in 98 of the 116 patients (84%), indicating that better LSC can be obtained in most patients using the SDP. Importantly, the ΔLSC values were significantly lower in the Child–Pugh class C group than in class A. In terms of imaging, ΔLSC was markedly higher in patients with visualized biliary excretion in HBP20 than in those without such visualization.

Additionally, we focused on patients with poor LSC in HBP20 and aimed to determine the factors contributing to the improvement in SDP. Univariate analysis revealed significant differences in total bilirubin levels, delayed time of SDP, visual contrast excretion into the bile duct during HBP20, and Child–Pugh classification. Multivariate analysis identified T-bilirubin and delayed time as independent factors contributing to an increase in LSC.

The effect of total bilirubin on LSC can be attributed to its pharmacokinetic relationship with gadoxetic acid. Gadoxetic acid is absorbed by the hepatocytes through the membrane transporters OATP1B1/1B3 and is primarily excreted into the capillary bile ducts via MRP2 [10, 21]. Interestingly, Pötter-Lang et al recently reported that using diluted gadoxetic acid in a 1:1 saline dilution with a slow injection rate of 1 mL/s enhanced liver contrast. They attributed this result to the increased length of time OATP1B1/1B3 has to pick up the contrast [22]. The activity of these transporters was decreased in rat models with cirrhotic livers [23]. Similarly, reduced hepatic contrast in HBP imaging was observed in patients with cirrhosis [12, 24]. Therefore, poor LSC indicates a decrease in liver function, which is related to a decrease in bilirubin excretion capacity, resulting in an increase in serum bilirubin levels.

In general, gadoxetic acid-enhanced MRI is not recommended in cases of severe hepatic dysfunction because adequate liver contrast in the HBP is not anticipated [9, 14, 21, 25]. However, considering its exceptional ability to detect hepatic nodular lesions, extending the application of gadoxetic acid-enhanced MRI to a larger number of patients may be beneficial. This study revealed that the addition of SDP could be beneficial for good LSC, even when HBP provided insufficient LSC in HBP20, through recirculation of gadoxetate disodium and the uptake of it into hepatocyte via OATP1B1/1B3 until it is excreted [22, 26]. Given the time and resources required to acquire SDP images, it is important to predict whether the LSC can exceed 1.5 in SDP. The optimal cut-off of total bilirubin for sufficient liver contrast on HBP has been reported to be around 1.75 mg/dL to 2.18 mg/dL [25]. To date, no reports have determined the cut-off value of total bilirubin for image quality improvement on SDP imaging. The analysis indicated that total bilirubin levels and delayed timing of SDP acquisition were significant predictors. Receiver operating characteristic (ROC) analysis established a total bilirubin cutoff value of 2.3, suggesting that values exceeding this threshold may reduce the effectiveness of SDP. Although not an independent predictive factor, visual contrast excretion into the bile duct during HBP20 is also a valuable imaging marker for predicting LSC exceeding 1.5 in SDP, which could be attributed to the pharmacokinetics of gadoxetic acid, as previously described [21]. Specifically, the visibility of the contrast agent in the bile ducts indicates that hepatocytes retain the ability to take up the contrast agent. In such instances, it can be considered that gadoxetic acid is taken up by the hepatocytes in a time-dependent manner.

An optimal LSC is essential for the effective detection of hepatic nodular lesions [11, 12]. In this study, we performed a Blind Image Interpretation test to assess the visibility of 61 nodules rated as LI-RADS category 4 or 5 in the group with poor LSC (LSC < 1.5). The findings revealed a significant improvement in nodule visibility, as evaluated by two independent readers blinded to the study (Reader 1: 2.92 ± 1.57 vs 3.79 ± 1.44; Reader 2: 2.34 ± 1.42 vs 3.36 ± 1.57; *p* < 0.001). We believe that this improvement in visibility is especially beneficial for the detection of early-stage HCC, which is typically small and visible only in the HBP on gadoxetic acid-enhanced MRI [8]. We believe that these findings can be considered a new approach to expand the indications for gadoxetic acid-enhanced MRI and to improve the detection of liver nodules and have considerable importance for future clinical applications.

Adopting a high flip angle in the HBP is another method to improve LSC. It can produce images with greater T1 emphasis compared to a low flip angle, thus enhancing the liver-lesion contrast [15–17]. Haradome et al reported that high flip angle imaging has higher sensitivity for detecting HCC compared to low flip angle imaging [27]. However, while high flip angle imaging can enhance the lesion-to-liver contrast, it might not be helpful when there is almost no contrast between the lesion and the liver in HBP20, a situation that is especially likely in cases of impaired liver function. Combining high flip angle imaging with SDP is an expected strategy to achieve better images for liver lesion detection in such cases.

This study had several limitations. First, introducing SDP imaging incurs both human and time-related costs. Second, the LSC assessment is not based on quantitative values. Because of the retrospective nature of the study, an ideal approach involving the use of a quantitative method such as the T1 map was not possible [14]. Third, our research did not dive deeply into the intricate mechanisms between LSC and the visualization of the contrast agent in the bile ducts. Finally, although our study determined that SDP imaging occurs between 60-min and 120-min post-injection, the optimal timing for SDP image acquisition remains ambiguous. In a clinical setting, we presume that performing one or two other examinations in between to achieve SDP imaging is rational; however, this assertion requires determining the optimal SDP timing by, for example, acquiring multiple additional SDP images and performing time-intensity curve analysis for each individual.

## Conclusion

In gadoxetic acid-enhanced MRI, the addition of SDP imaging improved the liver parenchymal contrast and enhanced lesion visibility, especially in patients with chronic liver disease and poor liver function. We believe that personalized scanning may be a better option to achieve better image quality for each individual.

## Supplementary information

ELECTRONIC SUPPLEMENTARY MATERIAL

## Abbreviations

CI: Confidence interval
HBP: Hepatobiliary-phase images
HBP20: Standard hepatobiliary phase images at 20 min post-contrast
HCC: Hepatocellular carcinoma
LSC: Liver-to-spleen contrast
ROC: Receiver operating characteristics
ROI: Regions of interest
SDP: Super delayed phase
