# Supplementary material for: Super delayed phase imaging in gadoxetic acid-enhanced MRI: investigating factors contributing to improved liver contrast
Source: Eur Radiol. 2024 Nov 29;35(6):3195–205. doi: 10.1007/s00330-024-11227-z (PMC12081474; doi:10.1007/s00330-024-11227-z)
Supplement: Supplementary file 1 — ELECTRONIC SUPPLEMENTARY MATERIAL [file 330_2024_11227_MOESM1_ESM.pdf]

Super Delayed Phase Imaging in Gadoxetic Acid-Enhanced MRI: Investigating Factors Contributing to Improved Liver Contrast
ELECTRONIC SUPPLEMENTARY MATERIAL

To further evaluate the relationship between acquisition time of SDP and LSC improvement, we redefined the acquisition times into three groups: 60-79 minutes, 80-99 minutes, and 100-120 minutes. We then performed ANOVA and Pearson correlation analyses on these groups.

Overall Improvement Rate: Out of all the SDP images, 54.4% (37/68) showed an improvement in image quality. This indicates that more than half of the scans benefited from some form of quality enhancement during the study.

ANOVA Results: An ANOVA test was conducted to determine if there were statistically significant differences in LSC improvement rates across the three new groups. The results indicated a significant difference between groups ($F(2, 65) = 3.391$, $p = 0.0397$). This suggests that the improvement in LSC varies significantly between the different acquisition time groups considered in this study.

Correlation Analysis: We also assessed the linear relationship between the mean acquisition time of each group and LSC improvement using Pearson's correlation coefficient. The analysis revealed a positive correlation between acquisition time and LSC improvement ($r = 0.302$, $p = 0.012$). This indicates that longer acquisition times are associated with higher rates of image quality improvement.

Limitations: It is important to note that in this study there is a single SDP timing in each individual case. In other words, the optimal timing of SDP images to best improve LSC is undetermined in this study. For this claim, the optimal SDP timing should be determined by acquiring multiple additional SDP images and performing a time-intensity curve analysis for each individual.

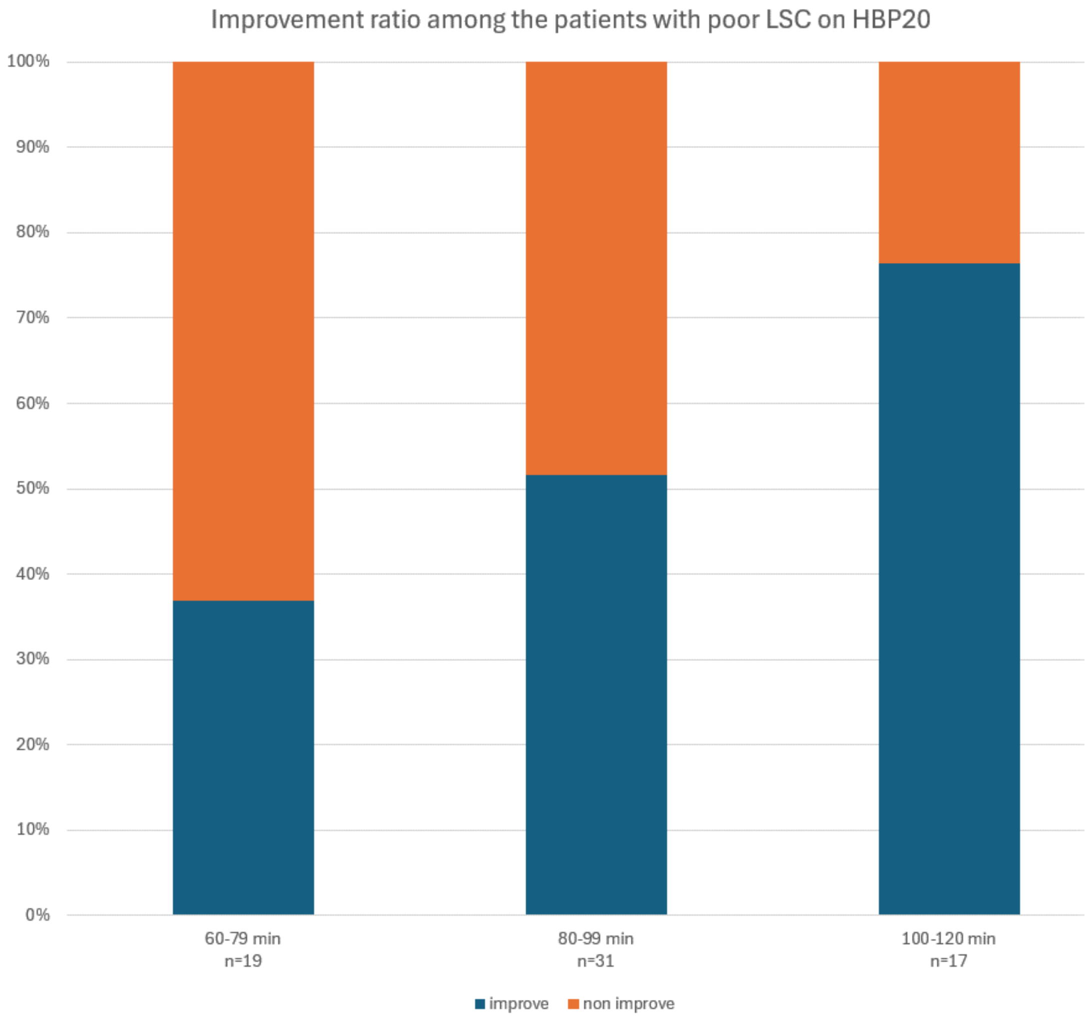

Eur Radiol (2024) Kobayashi T, Kozaka K, Matsubara T, et al.
